# QTL identification for seed weight and size based on a high-density SLAF-seq genetic map in peanut (*Arachis hypogaea* L.)

**DOI:** 10.1186/s12870-019-2164-5

**Published:** 2019-12-03

**Authors:** Shengzhong Zhang, Xiaohui Hu, Huarong Miao, Ye Chu, Fenggao Cui, Weiqiang Yang, Chunming Wang, Yi Shen, Tingting Xu, Libo Zhao, Jiancheng Zhang, Jing Chen

**Affiliations:** 1 Shandong Peanut Research Institute, Qingdao, 266100 People’s Republic of China; 2 Department of Horticulture, University of Georgia Tifton Campus, Tifton, GA 31793 USA; 3 State Key Laboratory for Crop Genetics and Germplasm Enhancement, Jiangsu Plant Gene Engineering Research Center, Nanjing Agricultural University, Nanjing, 210095 People’s Republic of China; 4 Institute of Industrial Crops, Jiangsu Academy of Agricultural Sciences, Nanjing, 210014 People’s Republic of China; 5 Qingdao Agricultural Radio and Television School, Qingdao, 266071 People’s Republic of China

**Keywords:** Peanut, SLAF-seq, High density genetic map, QTL, Seed weight, Seed size

## Abstract

**Background:**

The cultivated peanut is an important oil and cash crop grown worldwide. To meet the growing demand for peanut production each year, genetic studies and enhanced selection efficiency are essential, including linkage mapping, genome-wide association study, bulked-segregant analysis and marker-assisted selection. Specific locus amplified fragment sequencing (SLAF-seq) is a powerful tool for high density genetic map (HDGM) construction and quantitative trait loci (QTLs) mapping. In this study, a HDGM was constructed using SLAF-seq leading to identification of QTL for seed weight and size in peanut.

**Results:**

A recombinant inbred line (RIL) population was advanced from a cross between a cultivar ‘Huayu36’ and a germplasm line ‘6–13’ with contrasting seed weight, size and shape. Based on the cultivated peanut genome, a HDGM was constructed with 3866 loci consisting of SLAF-seq and simple sequence repeat (SSR) markers distributed on 20 linkage groups (LGs) covering a total map distance of 1266.87 cM. Phenotypic data of four seed related traits were obtained in four environments, which mostly displayed normal distribution with varied levels of correlation. A total of 27 QTLs for 100 seed weight (100SW), seed length (SL), seed width (SW) and length to width ratio (L/W) were identified on 8 chromosomes, with LOD values of 3.16–31.55 and explaining phenotypic variance (PVE) from 0.74 to 83.23%. Two stable QTL regions were identified on chromosomes 2 and 16, and gene content within these regions provided valuable information for further functional analysis of yield component traits.

**Conclusions:**

This study represents a new HDGM based on the cultivated peanut genome using SLAF-seq and SSRs. QTL mapping of four seed related traits revealed two stable QTL regions on chromosomes 2 and 16, which not only facilitate fine mapping and cloning these genes, but also provide opportunity for molecular breeding of new peanut cultivars with improved seed weight and size.

## Background

Peanut (*Arachis hypogaea* L.), an important source of edible oil and protein, is widely cultivated in more than 100 countries. The annual global peanut production increases rapidly in recent years resulting in a ten Mt elevation in yield from 2007 (37.51Mt) to 2017 (47.10Mt) (http://faostat.fao.org/) which paralleled to the constant increase in food demand [1]. Improving peanut yield through molecular breeding and optimized field management is amiable to the goals of sustainable agriculture. From the genetics point of view, peanut yield is influenced by a number of agronomic traits, such as height of main stem (HMS), total branch number (TBN), and the pod and seed/kernel traits [2]. Among these, 100 seed weight (100 SW), 100 pod weight and shelling percentage are important components of grain yield [2–4]. 100 SW is mainly determined by seed size which can be measured by seed length (SL) and seed width (SW) [2, 4]. Despite their contribution to yield, SL, SW and length to width ratio (L/W) are visual traits subject to selection during domestication and breeding [2]. In particular, shape of the peanut seeds (oblong or round) estimated by L/W is a critical factor determining the application of peanut varieties in the food processing factories. In China, round peanut is preferred for confectionary whereas oblong-shaped peanut is used for fried products. Identification of QTL/genes for seed related traits will advance our knowledge of biological pathways conditioning yield components and seed morphology.

Up to now, various genetic strategies to define QTLs or genes associated with seed traits have been conducted, including linkage mapping [2, 4], genome-wide association studies [5, 6] and bulked-segregant analyses [7]. High density genetic maps (HDGMs) provide essential information of linkage of genetic markers and facilitate QTL discovery [8, 9]. In the past decade, a number of genetic maps have been developed based on SSR markers for peanut. Over time, SSR based maps had increased marker density and map coverage [2, 3, 10–14]. However, genotyping by SSR markers is labor intensive and low throughput. Allelic SNP markers have the advantages of high frequency of occurrence in peanut genome [15, 16]. Combined with the next generation sequencing (NGS), a number of SNP-based genotyping technologies, especially SLAF-seq, have been applied to HDGM construction and QTL analysis in several species, such as sesame [17], soybean [18], cucumber [19, 20], cotton [21] and peanut [8, 22, 23]. In this study, a HDGM for cultivated peanut has been constructed based on SNP and SSR markers.

Compared with comprehensive studies on seed traits in rice [24, 25] and oilseed rape [26, 27], biological pathways controlling seed weight and size are not well understood in peanut. Up to now, QTL mapping for 100 seed weight, seed length, seed width and length to width ratio are still in progress. Using the bulked-segregant analysis (BSA), Gomez Selvaraj et al. [7] reported five SSR markers tightly linked to QTL regions for SL and 100SW. Fonceka et al. [28] identified several QTLs for pod and seed size that differentiated cultivated peanut from its wild relatives by using an advanced backcross population. Pandey et al. [5] performed genome wide association analysis by using 300 peanut genotypes, and identified 9 loci associated with SL, 3 with SW and 5 with 100SW. Using a F_2_ population, Huang et al. [29] successfully mapped QTLs for SL, SW, and 100SW, explaining phenotypic variance (PVE) from 1.69 to 17.88%. Chen et al. [4] utilized two F_2:3_ populations and detected 10 QTLs for SL and 7 for SW, with the PVE up to 20.80 and 14.43%, respectively. Chen at al [2]. conducted QTL mapping and meta-analysis with a RIL population, and reported 83 QTLs for pod- and seed-related traits. Wang et al. [8] constructed a SLAF-based HDGM from a RIL population and discovered two stable QTL regions for pod and seed related traits. Seed size QTL on chromosomes A05 and A07 were reported from two RIL populations [30, 31].

Due to the sequence similarity between the peanut diploid progenitors (*A. duranensis*, AA; *A. ipaensis*, BB) and cultivated tetraploid peanut (AABB), the genome sequences of the two peanut progenitors were informative in providing physical positions of genetic markers [12, 22, 32, 33]. However, homeologous recombination was identified in the newly released genome assemblies of cultivated peanut [34] suggesting erroneous assignment of QTL positions could occur by using the diploid genomes. Therefore, physical positions of QTL regions discovered in this manuscript were reported based on the cultivated peanut genome [34]. To further elucidate genomic regions conditioning seed related traits in peanut, we developed a recombinant inbred line (RIL) population for QTL mapping. The parental genotypes, a large-seeded cultivar ‘Huayu36’ and a small-seeded germplasm line ‘6–13’, were characterized by contrasting phenotypes in seed weight, seed size and shape. SLAF-seq and SSR analysis were conducted to generate sufficient markers for HDGM construction. QTL mapping of four seed related traits collected from four environments revealed two stable QTL regions on chromosomes 2 and 16, which may facilitate the peanut breeding with improved seed characteristics.

## Results

### Phenotyping of the parents and RIL individuals.

**Fig. 1:**
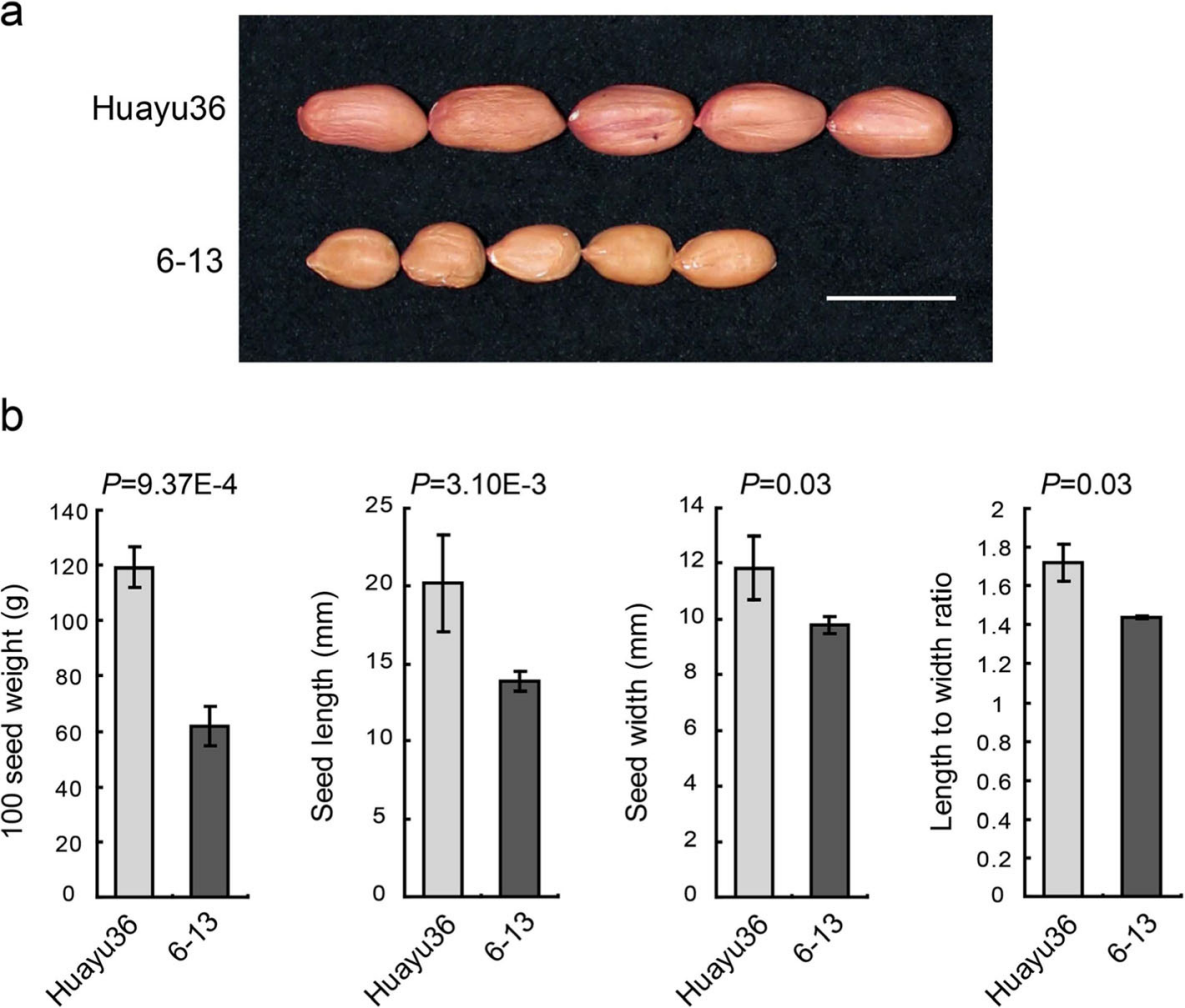
Phenotypic characterization of seeds from ‘Huayu36’ and ‘6-13', (**a**), Seed morphology of two parents ‘Huayu36’ and ‘6-13’. Scale bar: 2 cm. (**b**), Comparisons of 100 seed weight, seed length, seed width and length to width ratio between ‘Huayu36’ and ‘6–13′. Data shown as mean ± s.e.m. (*n* = 9). Student’s t-test was used to generate the *P* values

**Fig. 2:**
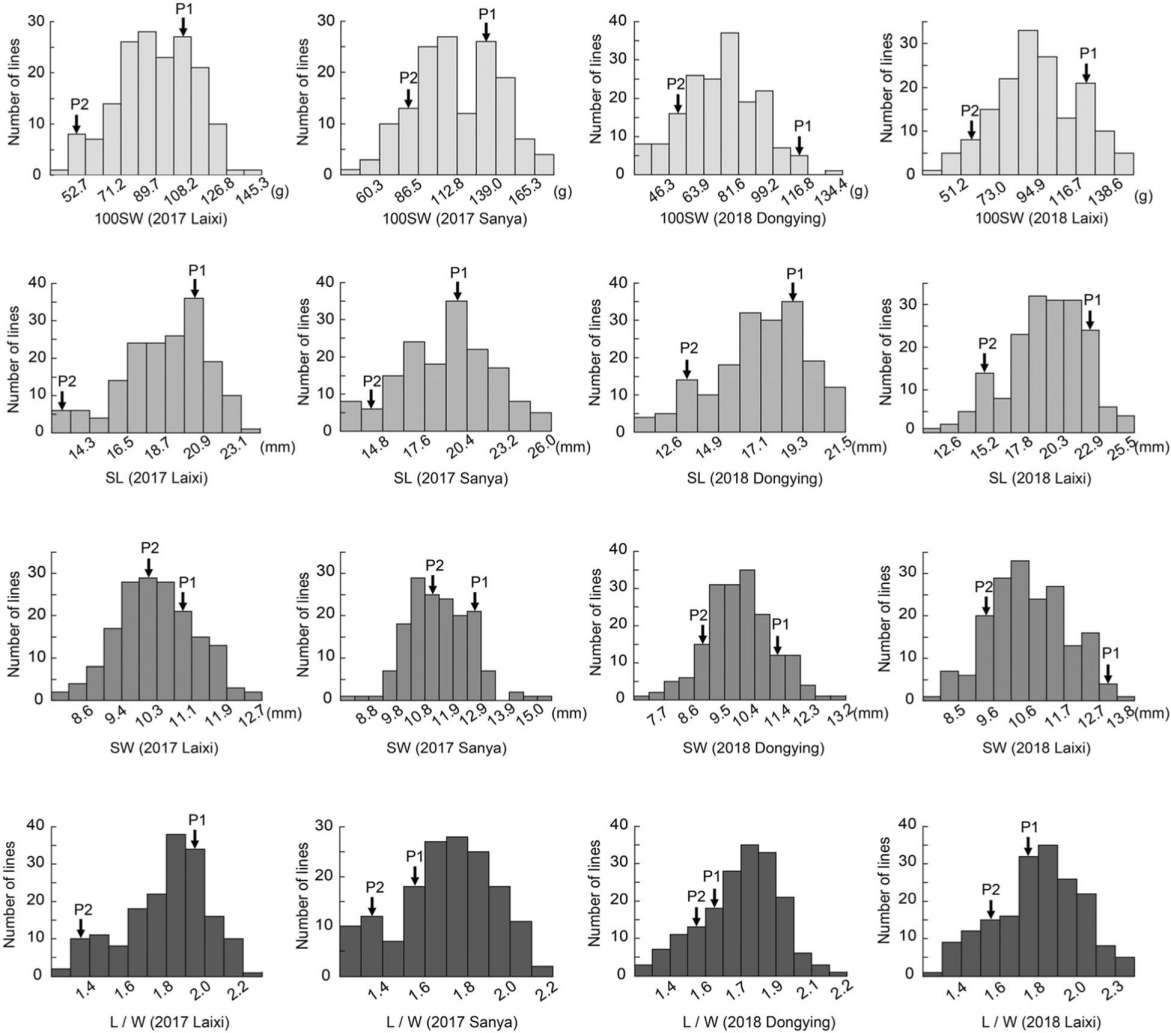
Phenotypic distribution of seed traits for the RIL population, The x-axis shows the range of seed traits, including 100 seed weight(100SW), seed length(SL), seed width (SW) and length-width ratio (L/W) in four environments (2017 Laixi, 2017 Sanya, 2018 Dongying and 2018 Laixi). The y-axis shows the number of individuals of the RIL population. P1 and P2 represent the parents ‘Huayu36’ and ‘6–13’, respectively

To identify novel QTL/genes modulating seed traits in peanut, a RIL population consisting of 181 individuals was created from a cross between ‘Huayu36’ and ‘6–13’. The maternal parent ‘Huayu36’ was a large-seeded cultivar (Fig. 1a), with 100SW, SL, SW and L/W up to 119.30 ± 7.17 g, 20.15 ± 3.12 mm, 11.83 ± 1.13 mm and 1.72 ± 0.10, respectively (Fig. 1b). The paternal parent ‘6–13’ was a germplasm line with significantly smaller seed weight and size (Fig. 1a). The corresponding measurements for ‘6–13’ were 61.83 ± 7.28 g, 13.89 ± 0.63 mm, 9.80 ± 0.30 mm and 1.44 ± 0.03 (Fig. 1b). The RIL population and its parents were planted in four environments (Laixi, 2017; Sanya, 2017; Dongying, 2018; Laixi, 2018) and phenotypic data for the four seed related traits (100SW, SL, SW and L/W) demonstrated normal distribution among the population (Fig. 2; Table 1). Transgressive segregation was observed in most environments (Fig. 2), indicating polygenic inheritance of the measured traits. ANOVA (analysis of variance) results indicated that the effects of genotypes (G), environments (E), and interaction of G and E (G × E) were all significant for all measured traits except for G × E for SL (Table 2). All four seed traits exhibited relatively high broad-sense heritability (*h*^*2*^), ranging from 0.77 to 0.89 (Table 2), which suggested genetics plays a major role in controlling seed size and weight, yet the environmental influence should not be ignored. Pairwise correlation analysis indicated significant positive correlation between 100SW and SL (*r* = 0.793) as well as between 100SW and SW (*r* = 0.722). A positive correlation was found between SL and SW (*r* = 0.537). L/W was positively correlated with both 100SW (*r* = 0.435) and SL (*r* = 0.809), yet no significant correlation was found between with SW and L/W (Table 3).

**Table 1:** Phenotypic variation of seed traits among the RIL population in four environments

Trait	Environment	Mean ± SD^a^	Min^b^	Max^.c^	Skew	Kurt	Sig. of K-S test^d^
100SW	2017 Laixi	89.86 ± 20.22	43.12	139.08	−0.171	−0.491	0.200
	2017 Sanya	112.81 ± 28.89	46.46	174.20	−0.032	− 0.614	0.200
	2018 Dongying	72.65 ± 19.20	30.09	125.98	−0.011	−0.349	0.200
	2018 Laixi	94.93 ± 23.96	39.80	148.30	0.043	−0.468	0.200
SL	2017 Laixi	18.74 ± 2.41	12.62	23.17	−0.538	−0.209	0.035*
	2017 Sanya	19.03 ± 3.08	12.27	25.98	−0.085	− 0.332	0.096
	2018 Dongying	17.08 ± 2.43	10.47	21.37	−0.556	− 0.287	0.001**
	2018 Laixi	19.06 ± 2.82	11.11	25.12	−0.461	−0.065	0.078
SW	2017 Laixi	10.27 ± 0.91	7.99	12.55	0.042	−0.387	0.200
	2017 Sanya	11.35 ± 1.14	8.11	14.99	0.247	0.370	0.200
	2018 Dongying	998 ± 1.00	7.13	12.81	0.052	0.251	0.200
	2018 Laixi	10.60 ± 1.15	7.80	13.46	0.036	−0.443	0.200
L/W	2017 Laixi	1.84 ± 0.22	1.29	2.30	−0.506	0.412	0.000**
	2017 Sanya	1.69 ± 0.23	1.20	2.19	−0.300	− 0.467	0.200
	2018 Dongying	1.72 ± 0.18	1.25	2.18	−0.461	−0.065	0.000**
	2018 Laixi	1.82 ± 0.25	1.25	2.38	−0.236	− 0.331	0.002

^a^SD, standard deviation;

^b^Min, minimum value;

^c^Max, maximum value;

^d^Sig of K-S test, significance for normality test by Kolmogorov-Smirnov;

* and ** mean significant at *P* < 0.05 and *P* < 0.01, respectively

**Table 2:** Analysis of the broad-sense of heritability of four seed related traits

Traits	Source	DF^a^	SS^b^	MS^c^	*F* value	*P*	*h*^*2*^
100SW	G	178	525,776.44	2953.80	1326.45	< 0.01	0.89
	E	3	248,989.34	82,996.45	37,270.95	< 0.01	
	G × E	454	161,141.02	354.94	159.39	< 0.01	
	Error	632	1407.36	2.2268			
SL	G	180	8045.98	44.70	12.34	< 0.01	0.83
	E	3	1116.56	372.19	102.77	< 0.01	
	G × E	495	4029.50	8.14	2.25	> 0.05	
	Error	675	2444.63	3.62			
SW	G	180	1186.23	6.59	7.58	< 0.01	0.77
	E	3	348.60	116.20	133.59	< 0.01	
	G × E	495	843.57	1.70	1.96	< 0.05	
	Error	675	587.11	0.87			
L/W	G	180	58.82	0.33	6.96	< 0.01	0.81
	E	3	4.31	1.46	30.58	< 0.01	
	G × E	495	33.52	0.07	1.44	< 0.01	
	Error	675	31.68	0.05			

^a^*DF* degree of freedom;

^b^*SS* sum of square;

^c^*MS* mean of square

**Table 3:** Pearson’s correlation analysis among the measured traits of the RIL population

Traits	100SW	SL	SW	L/W
100SW	–	^b^	^b^	^b^
SL	0.793	–	^b^	^b^
SW	0.722	0.537	–	n.s.^a^
L/W	0.435	0.809	−0.055	–

^a^ n.s not significant at *P* < 0.05;

^b^ indicated significant at *P* < 0.01

### SLAF sequencing, SNP and SSR genotyping.

A total of 327.08 Gb raw sequencing data containing 1635.75 M reads was obtained after sequencing both parents and 181 RIL lines, of which 9.70 Gb data with 48.51 M reads was from the maternal line ‘Huayu36’, and 7.56 Gb data with 37.82 M reads was from the paternal line ‘6–13’, respectively (Table 4; Additional file 2: Table S5). The GC (percentage of guanine and cytosine in all four bases) content was 37.66%, and Q30 ratio (bases with a quality score of 30, indicating 99% confidence) was 94.66% on average (Additional file 2: Table S5). After discarding the low quality reads, 1,614,182 SLAF tags containing 510,204 SNPs were mined based on the Tifrunner reference genome, of which 733,610 and 693,570 SLAFs were identified from maternal and paternal parents with the sequencing depth of 50.91- and 52.33-fold, respectively (Table 4; Additional file 2: Table S6). The number of SLAFs in the F_6_ progenies was 506,417, with an average coverage of 16.13-fold, corresponding to 8,207,746 reads (Additional file 2: Table S6).

**Table 4:** Summary of SLAF-seq data for the RIL population

Total reads	
Number of reads	1635.75M^a^
Number of reads in high quality	1541.04 M
SLAF^b^ tags	
Number of SLAFs	1,614,182
Average depth of SLAFs in parents	51.62
Average depth of SLAFs in individuals	16.13
SNP^c^ markers detected in SLAF tags	
Number of SNPs	510,204
Average number of SNPs in parents	363,559
Average number of SNPs in individuals	293,244
Number of polymorphic SNPs	12,950
High-quality SNP markers	
Number of high-quality SNP markers	3829
Average depth in parents	95.18
Average depth in individuals	23.30

^a^ *M* million;

^b^ *SLAF* specific locus amplified fragment;

^c^ *SNP* single nucleotide polymorphism

A total of 510,204 SNPs were obtained in the SLAF tags, and 12,950 were successfully encoded as polymorphic with a polymorphism rate of 2.54% (Table 4). According to the genotype encoding rule, the polymorphic SNPs were grouped in different segregation patterns (ef × eg, hk × hk, lm × ll, nn × np, aa×bb, ab×cc, cc × ab, ab×cd). Since the RIL population was derived from a cross between two homozygous genotypes, a total of 6124 SNPs belonging to aa×bb pattern were extracted (Additional file 2: Figure S1). After filtering the low-quality SNPs, 3829 were available for linkage analysis (Table 4).

### Construction of the high-density genetic map

**Fig. 3:**
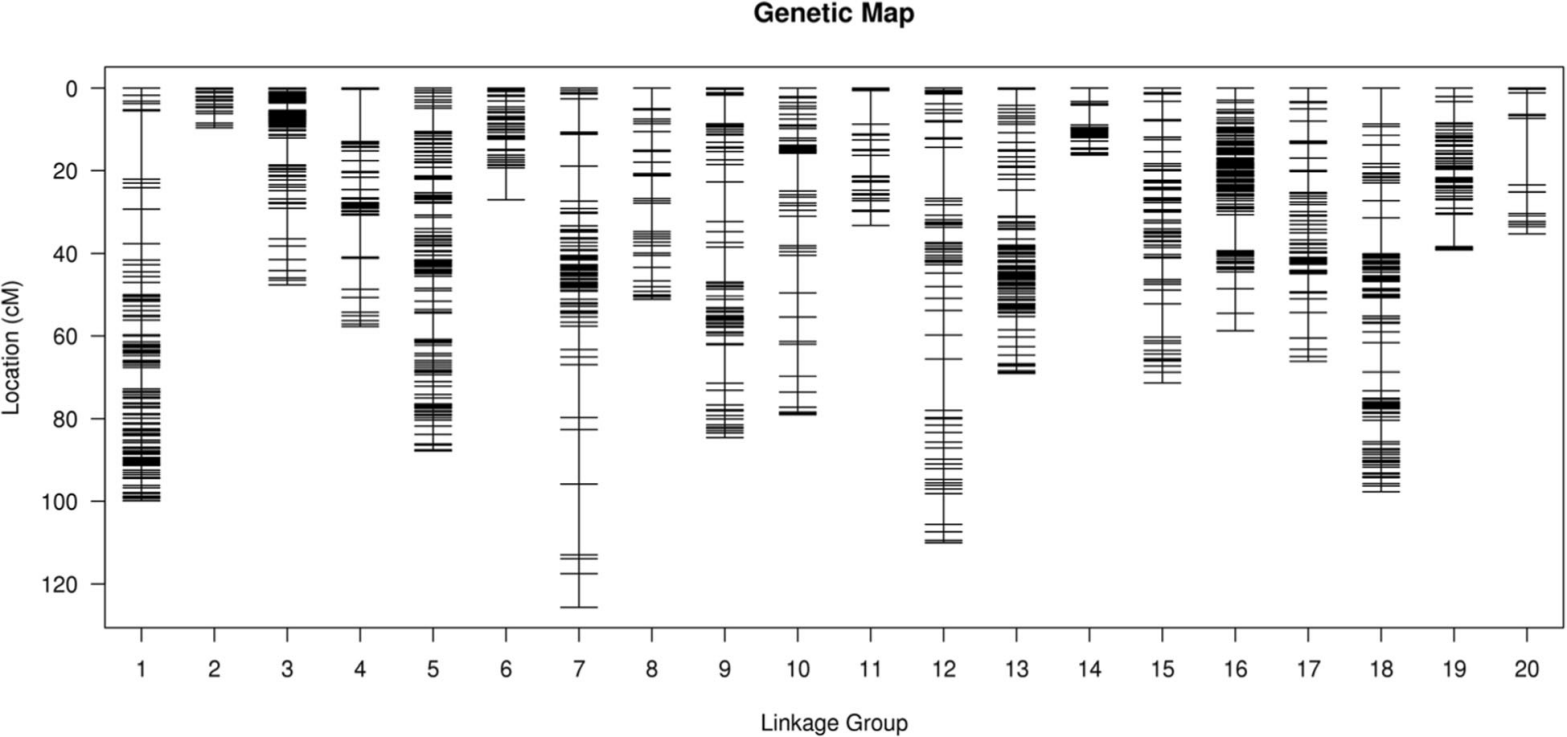
High density genetic map of the RIL population using SNP and SSR markers, The markers were indicated by black bars. The x-axis represents 20 linkage groups and y-axis represents genetic distance

For the HDGM construction, 3866 markers (3829 SNPs and 37 SSRs) were assigned to 20 linkage groups (LGs) (Fig. 3; Table 5). This map covered a total of 1266.87 cM genetic distance ranging from 9.61–125.63 cM for each linkage group with average marker interval of 0.33 cM (Table 5). LG 7 was the longest group covering a distance of 125.63 cM with 153 loci, while LG 2 was the shortest group spanning 9.61 cM with 39 loci. LG 3 hosted 421 loci which was the highest among all linkage groups, whereas LG 20 had only 22 loci, the least among the linkage groups (Table 5). In addition, the 37 SSRs were distributed across 16 LGs, with no SSRs assigned on LG 6, LG 11, LG 12 and LG 20 (Table 5). Subsequently, the degree of the map uniformity and inter-marker linkage were evaluated by the percentage of ‘Gaps≤5 cM’, which ranged from 85.71 to 100% with an averaged value of 97.04%. The largest gap existed on LG 7, which was 17.05 cM (Table 5).

**Table 5:** Summary of the high-density genetic map

LG^a^	Total marker	Total distance(cM)	Average distance of adjacent markers (cM)	Largest gap (cM)	Gaps≤5 cM (%)	SSR^b^
1	280	99.88	0.36	16.6	98.57	6
2	39	9.61	0.25	2.33	100.00	1
3	421	47.58	0.11	7.33	99.52	4
4	352	57.73	0.16	12.67	99.15	2
5	289	87.81	0.30	6.36	99.31	3
6	68	27.05	0.40	7.70	99.51	0
7	153	125.63	0.82	17.05	94.74	2
8	47	51.13	1.09	6.82	95.65	1
9	317	84.58	0.27	9.66	98.73	2
10	64	79.02	1.23	9.13	90.48	1
11	34	33.24	0.98	8.12	93.94	0
12	74	110.01	1.49	12.37	93.15	0
13	313	68.96	0.22	6.36	99.68	2
14	261	16.24	0.06	4.86	100.00	2
15	259	71.35	0.28	8.05	99.22	2
16	369	58.75	0.16	8.64	99.46	1
17	100	66.17	0.66	6.16	97.78	2
18	270	97.74	0.36	8.70	98.51	3
19	134	39.15	0.29	7.75	98.50	3
20	22	35.24	1.60	16.12	85.71	0
Total	3866	1266.87	0.33	17.05	97.04	37

^a^*LG* linkage group;

^b^*SSR* simple sequence repeat

To assess the quality of the HDGM, we conducted colinearity analysis by comparing the genetic positions of markers on each LG to their physical positions. Despite three obvious inverted segments on LG 8 and LG 17, a relative high colinearity between the genetic and genomic positions was displayed (Additional file 2: Figure S2), confirming a well ordered marker assignment.

### QTL identification for seed related traits

**Fig. 4:**
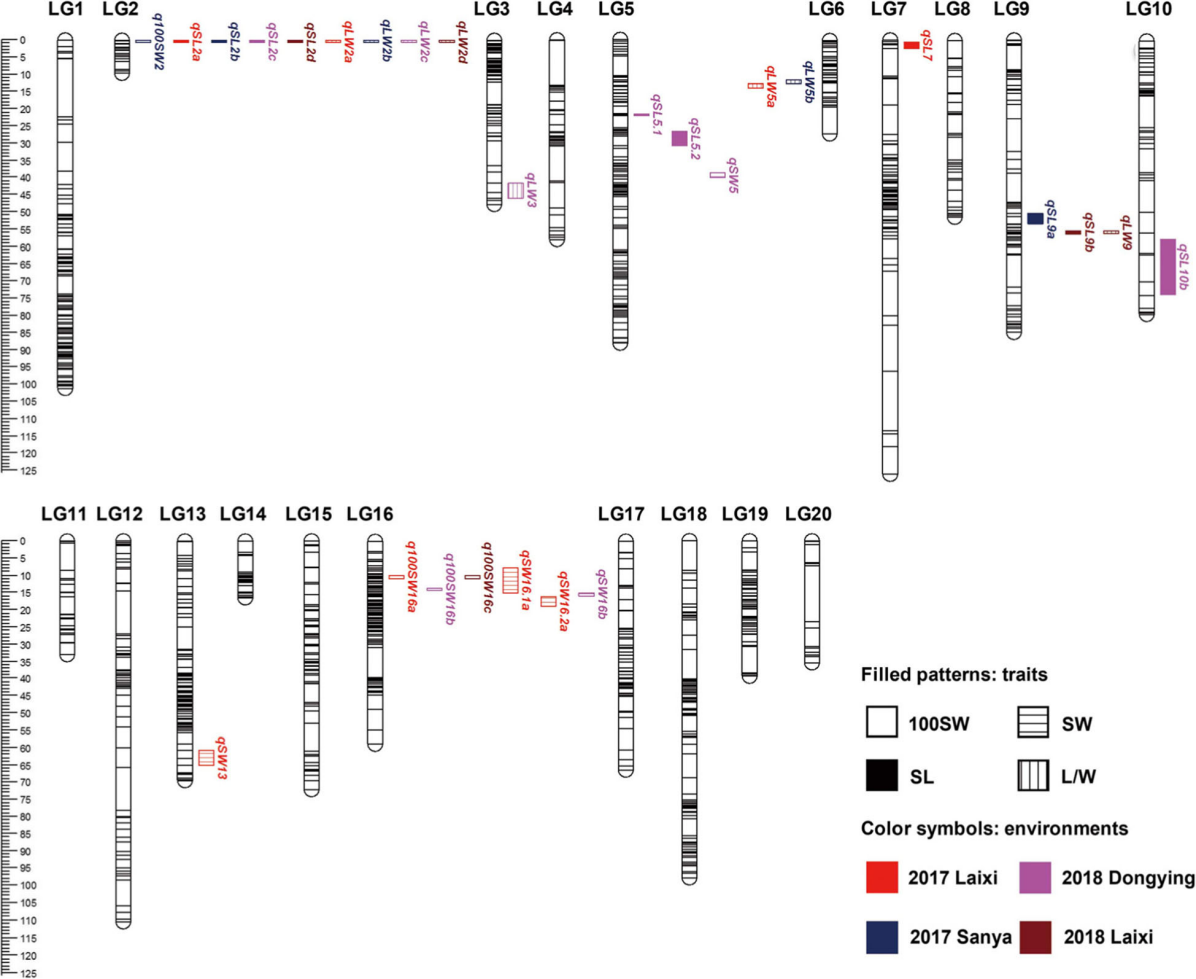
Seed related QTLs detected in four environments

QTL mapping resulted in identification of 27 QTLs for all 4 seed related traits, with the LOD values of 3.16–31.55 and explaining phenotypic variation (PVE) from 0.74 to 83.23% (Fig. 4; Table 6).

**Table 6:** QTL analysis for four seed related traits

Trait	Env^a^	QTL	LG^b^	CI^c^	Flanking Markers	Physical position (Mb)	LOD^d^	ADD ^e^	PVE ^f^ (%)
100SW	2017LX	*q100SW16a*	16	9.8–10.7	Marker9375–Marker9395	8.84–11.61	3.34	−11.43	29.81
	2017SY	*q100SW2*	2	0–0.5	Marker938–Marker893	92.75–99.81	6.27	−14.87	24.69
	2018DY	*q100SW16b*	16	13.6–14.1	Marker9444–Marker9463	15.96–18.31	7.60	−11.83	35.39
	2018LX	*q100SW16c*	16	9.9–10.7	Marker9372–Marker9395	8.49–11.61	5.96	−13.70	30.47
SL	2017LX	*qSL2a*	2	0–0.7	Marker938–Marker893	92.75–99.81	17.62	−1.96	61.74
	2017LX	*qSL7*	7	0.6–2.6	Marker4618–Marker4664	74.27–76.91	3.16	0.21	0.74
	2017SY	*qSL2b*	2	0–0.8	Marker938–Marker893	92.75–99.81	15.88	−2.08	42.43
	2017SY	*qSL9a*	9	50.3–53.2	Marker5024–Marker5026	19.18–19.29	3.55	0.64	4.05
	2018DY	*qSL2c*	2	0–0.8	Marker938–Marker893	92.75–99.81	8.38	−1.80	51.20
	2018DY	*qSL5.1*	5	21.6–22.1	Marker3540–Marker3535	93.27–94.03	3.21	−1.60	40.25
	2018DY	*qSL5.2*	5	26.7–30.8	Marker3455–Marker3523	85.42–91.99	3.41	−1.53	36.91
	2018DY	*qSL10*	10	57.4–73.3	Marker5972–Marker6000	108.15–115.70	3.61	−1.32	27.48
	2018LX	*qSL2d*	2	0–0.7	Marker938–Marker893	92.75–99.81	20.19	−2.13	53.60
	2018LX	*qSL9b*	9	55.2–56.2	Marker5044–Marker5514	22.03–90.20	3.75	0.95	9.62
SW	2017LX	*qSW13*	13	60.3–64.7	Marker7532–Marker7533	138.32–138.50	3.45	−0.32	12.12
	2017 LX	*qSW16.1a*	16	7.7–14.8	Marker9360–Marker9483	7.33–19.54	4.17	−0.35	13.68
	2017LX	*qSW16.2a*	16	16.1–18.6	Marker9525–Marker9662	25.49–53.24	3.62	−0.33	12.64
	2018DY	*qSW5*	5	38.5–40.0	Marker3468–Marker3409	77.32–86.33	3.92	−0.40	15.07
	2018DY	*qSW16b*	16	14.8–15.8	Marker9464–Marker9503	18.81–22.71	4.47	−0.48	21.58
L/W	2017LX	*qLW2a*	2	0–0.7	Marker938–Marker893	92.75–99.81	27.47	−0.21	83.23
	2017LX	*qLW5a*	5	12.9–14.0	Marker3612–Marker3590	102.71–106.10	4.18	−0.15	43.66
	2017SY	*qLW2b*	2	0–0.6	Marker938–Marker893	92.75–99.81	15.41	−0.19	65.77
	2017SY	*qLW5b*	5	11.7–12.9	Marker3622–Marker3604	105.45–107.08	4.3	−0.15	45.83
	2018DY	*qLW2c*	2	0–0.7	Marker938–Marker893	92.75–99.81	24.52	−0.15	69.07
	2018DY	*qLW3*	3	41.5–45.8	Marker1886–Marker1906	140.53–143.23	4.9	0.04	4.85
	2018LX	*qLW2d*	2	0–0.7	Marker938–Marker893	92.75–99.81	31.55	−0.21	70.64
	2018LX	*qLW9*	9	55.3–56.0	Marker5041–Marker5511	21.40–89.29	4.39	0.09	4.47

^a^ *Env* environment;

^b^ *LG* linkage group;

^c^ *CI* confidence interval;

^d^ *LOD* logarithm of the odds;

^e^ *ADD* additive effect;

^f^ *PVE* phenotypic variation explained

For 100SW, a total of 4 QTLs were detected on two chromosomal regions (Fig. 4), explaining phenotypic variation of 24.69–35.39% (Table 6). One consistent QTL region was detected in more than one environment, and located in the marker interval Marker9375–Marker9395 on LG 16 spanning 2.77 Mb which was detected in Laixi 2017 and Laixi 2018 with the PVE of 29.81 and 30.47%, respectively (Table 6). Although *q100SW16b* (PVE = 35.39%) was also identified on LG 16, the QTL position was shifted lower than the first consistent QTL region. *Q100SW2* was identified in only one environment, with a PVE of 24.69% (Fig. 4; Table 6).

For SL, a total of 10 QTLs were mapped on LGs 2, 5, 7, 9 and 10 (Fig. 4). *QSL2* was a major consistent QTL region (Marker938–Marker893) spanning 7.06 Mb on LG 2 with the PVE up to 61.47% (Table 6). This QTL region was identified in all four environments. Two major QTLs (*qSL5.1* and *qSL5.2*) were tightly linked on LG 5 and identified in the same season, with PVE of 40.25 and 36.91%, respectively. Another QTL, *qSL9*, was identified in the Sanya_2017 and Laixi_2018 datasets with PVE of 4.05 and 9.62%, respectively (Fig. 4; Table 6). Both *qSL7* and *qSL10* were identified in only one season. Except for *qSL9*, all the alleles from ‘Huayu36’ increased measurements of seed traits (Table 6).

For SW, 5 QTLs were identified, accounting for 12.12–21.58% PVE (Fig. 4; Table 6). Three major QTLs (*qSW16.1a*, *qSW16.2a*, *qSW16*b) were mapped on LG 16 (Fig. 4), with the ‘Huyu36’ allele contributing to increased SW (Table 6). *QSW16.1a* (PVE 13.68%) and *qSW16.2a* (PVE 12.64%) were detected in the same environment, while *qSW16b* (PVE 21.58%) was identified in a different season (Table 6). The position of *qSW16.1a* and *qSW16*b overlapped with the consistent QTL region detected for 100SW. Another two major QTLs for SW (*qSW5* and *qSW13*) were detected on LGs 5 and 13 and accounted for PVE of 15.07 and 12.12%, respectively (Table 6). These QTLs were detected in only one environment.

For the L/W, 8 associated QTLs were mapped on LGs 2, 3, 5 and 9 (Fig. 4; Table 6). Among these, the consistent QTL region on LG 2 was detected in all four environments (PVE = 65.77 to 83.23%), and co-localized with the QTL region identified for SL. Donor alleles for increased trait measurements came from ‘Huayu36’. Another consistent QTL region (*qLW5a* and *qLW5b*) was detected on LG 5, accounting for 43.66 and 45.83% PVE, respectively (Table 6). Two minor QTLs, *qLW3* (PVE 4.85%) and *qLW9* (PVE 4.47%) were identified, with the ‘6–13’ allele contributing to the L/W trait (Table 5). Additionally, *qLW9* overlapped with the region detected for *qSL9b* (Fig. 4).

### Functional annotation of two stable and pleiotropic QTL regions.

**Fig. 5:**
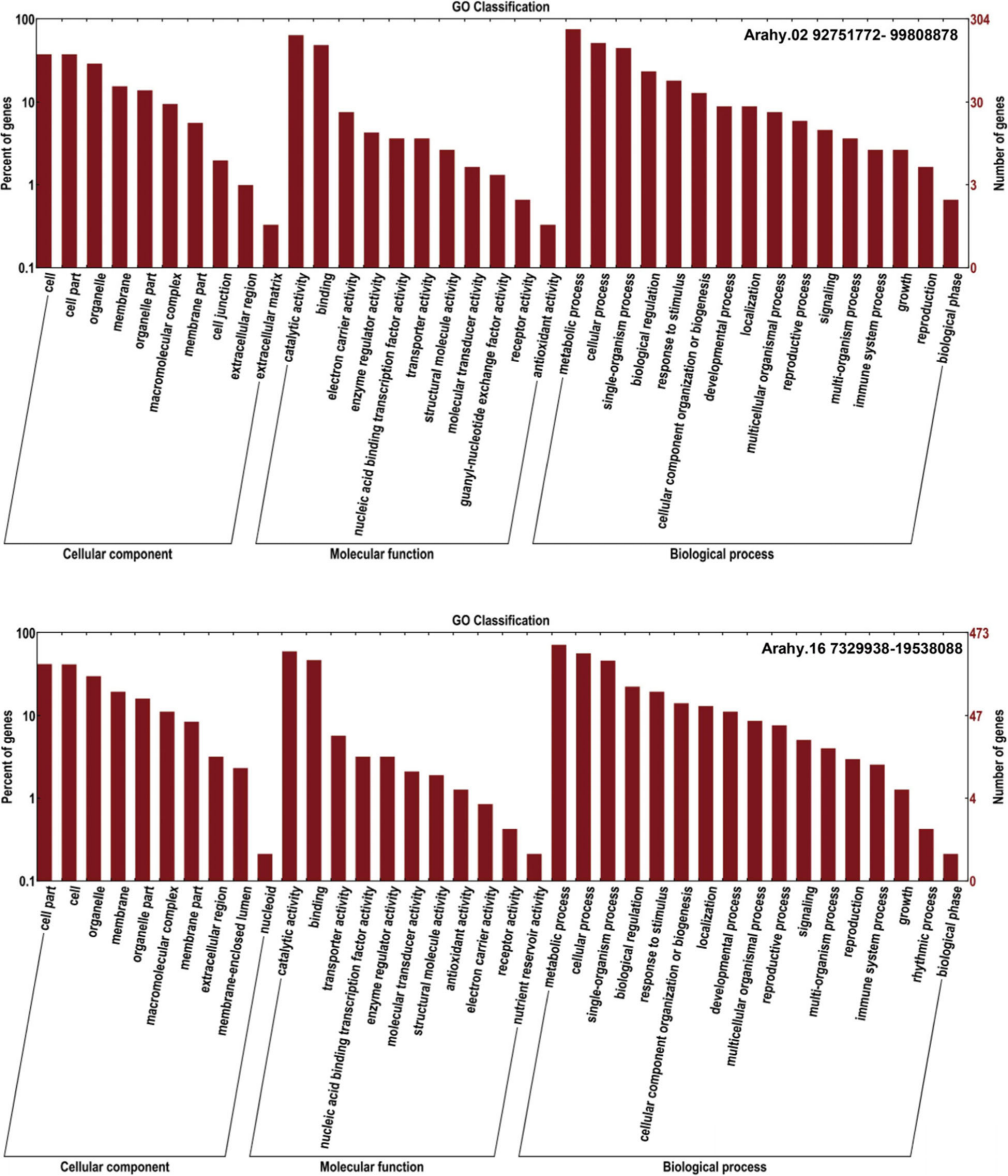
GO annotation of genes within the region I and II on chromosome 2 and 16 respectively

In order to reveal genes/genetic pathways potentially conditioning seed size, genes within the two consistent QTL regions on chromosomes 2 and 16 were extracted from the Tifrunner reference genome for annotation. The first QTL region (defined as the region I) on chromosome 2, with flanking markers Marker938–Marker893, spanned a genomic distance of 7.06 Mb, and contained 514 candidate genes by Nr database (Additional file 3: Table S7). GO annotation showed that the majority of genes had specific functional assignment: the cell (114), cell part (114) and organelle (88) in the cellular component category; catalytic activity (195), binding (148) and electron carrier activity (23) in the molecular function category; metabolic process (230), cellular process (157) and single-organism process (136) in the biological process category (Fig. 5). The other QTL region (defined as the region II) on chromosome 16, with flanking markers Marker9360–Marker9483 covered a genomic distance of 12.21 Mb. A total of 684 candidate genes were obtained within this region (Additional file 3: Table S8), among which the majority terms were cell (199), cell part (197) and organelle (142) for cellular component category; catalytic activity (195), binding (148) and transporter activity (27) for molecular function category; metabolic process (340), cellular process (267) and single-organism process (218) for the biological process category (Fig. 5).

A total of 66 SNPs were detected in these two QTL regions (16 SNPs in the region I and 50 SNPs in the region II), which were mostly located in intergenic regions or resulted in synonymous mutation (Additional file 3: Table S9). Phytohormones such as brassinosteroid (BR) were known to play crucial roles in seed development [35]. Deficiency in BR synthesis and signal transduction pathway leads to off-type exhibitions in seed size and mass [36–39]. In the region I, three candidate genes arahy.T43K8I.1, arahy.T43K8I.2 and arahy.T43K8I.3 were all homologous to the BSU1 (BRI1 suppressor 1) (Additional file 2: Figure S3; Additional file 3: Table S7), which was reported to function in brassinosteroid (BR) signaling and affect plant growth [40, 41]. Meanwhile, a candidate gene arahy.BC5R4P.1 encoding the putative auxin response factor 2 (ARF2)–like protein was highlighted in the region II (Additional file 2: Figure S3; Additional file 3: Table S8), of which the homolog acted downstream of BZR1 and BR signal in regulating seed size in *Arabidopsis* [42].

## Discussion

### A SLAF based HDGM for cultivated peanut.

In this study, two parental genotypes with contrasting seed characteristic were selected to develop a RIL population. High levels of phenotypic variation among the RIL lines allowed for the detection of QTL using the HDGM. Previously, the narrow genetic basis of cultivated peanut resulted in an extremely low degree of polymorphism in various forms of molecular markers, which restrains the construction of HDGM in peanut [13, 43, 44]. The SLAF-seq strategy, a combination of locus-specific amplification and next generation sequencing has been successfully applied in large-scale SNP mining in various species, revealing its mature pipelines and advantages in HDGM construction and favorable QTL identification [17, 18, 21, 45]. Currently, three HDGMs based on SLAF-seq have been published in cultivated peanut, all of which consisted of more than 2000 high-quality SNP markers, and resulted in yield and oil quality related QTL detection in cultivated peanut [8, 22, 23]. In this study, close to 4000 markers were placed on the linkage map allowing for fine genetic mapping of traits of interest (Table 5). Meanwhile, colinearity analysis validated the high quality of this map. The present SLAF-based HDGM was constructed based on the newly published genome of tetraploid cultivated peanut (*A. hypogaea* cv. Tifrunner) [34], rather than the reference genomes of two ancestral diploid species (*A. duranensis*, AA; *A. ipaensis*, BB) [32, 33]. Although sequence conservation between the diploid and tetraploid species was high, tetrasomic events and chromosome inversions occurred after the polyploidization event forming the cultivated peanut species [34, 46–49]. Thus, using the cultivated peanut reference genome reduced the risk of incorrect assignment of marker positions and improved the accuracy of QTL/genes identification.

### QTL identification for seed weight and size.

The complex genetic basis of seed weight and size has been well characterized in crops, which were controlled by a number of genes involved in different pathways [25, 27, 50, 51]. However, the underlying genetic mechanism for peanut seed related traits is poorly understood and needs further investigation. Currently, several seed related QTLs have been identified with variable PVE and chromosomal positions from different parental genotypes [2, 4, 8, 28, 29]. And the previously identified QTL for 100SW, SL and SW were widely distributed on various LGs, indicating complex polygenic inheritance of such traits. Similarly, in the present study, a total of 27 QTLs for 4 seed related traits were detected on chromosomes 2, 3, 5, 7, 9, 10, 13 and 16, explaining phenotypic variation from 0.74 to 83.23% (Fig. 4; Table 6). Among these, *qSL2*, *qLW2* and *q100SW16* with more than 29.81% PVE were consistently detected in at least three environments, indicating stable genetic effects across environments. There were 5 and 3 QTLs covering two genomic regions on chromosomes 5 and 9, which contained three transposable element markers (AhTE0523, AhTE0278 and AhTE0437) previously associated with pod size [6]. Meanwhile, the QTL region covering 29.7 Mb on chromosome 5 agreed with the location of QTL for pod weight detected by Hake et al. [54].

The co-localization of *qSL2* and *qLW2* on chromosome 2 and *q100SW16* and *qSW16* on chromosome 16 was in agreement with the high positive correlation within each pair of traits. It was often observed that yield related QTLs exhibited pleiotropic effects on more than one trait [2, 53, 55]. Application of genetic markers within these QTL regions in breeding programs could potentially optimize the selection of multiple seed related traits. Previous studies have reported several yield related QTLs on B06 [2, 8, 28]. Among these, Wang et al. [8] identified one QTL region on B06 with physical coverage of 119.8 Mb–128.8 Mb, which was distant from QTL regions reported here (physical coverage: 7.33 Mb–21.71 Mb and 25.49 Mb–53.24 Mb). Chen et al. [2] reported three QTL regions for both 100SW and SW on B06, among which two (physical coverage: 10.6 Mb–21.6 Mb and 12.2 Mb–74.9 Mb) overlapped with our results, suggesting a possibly similar genetic basis within these regions. Fonceka et al. [28] identified one marker (TC3H07_B) on B06, associated with seed weight only under water-limited condition. Since the physical position of TC3H07_B is unknown, we are unable to determine whether this marker locates in our detected QTL regions. Nevertheless, to our knowledge, the region I on chromosome 2 is novel (Fig. 4), since no related QTL has been reported on this chromosome yet.

### Functional annotation in two stable pleiotropic QTL regions.

Functional annotation was provided for 514 and 684 genes within the QTL regions I and II, facilitating the understanding of their putative biological functions. A total of 66 SNPs were detected in the region I and II, none of which led to alteration in protein coding sequences (Additional file 3: Table S9). The insufficient marker density limited the prediction of target genes. However, these SNPs might be used to design KASP (kompetitive allele-specific polymerase chain reaction) markers for fine mapping and facilitating molecular breeding [56].

Previous studies on other species provided useful information to understand the putative mechanisms for peanut seed size regulation [25–27, 50–53]. Hormones have been widely demonstrated to function in seed development, among which BR plays key roles in seed size regulation [36–39]. In the region I, three genes arahy.T43K8I.1, arahy.T43K8I.2 and arahy.T43K8I.3 encoded proteins homologous to the BSU1 (Additional file 2: Figure S3; Additional file 3: Tables S7, S8), a key factor in fine tuning the BR responses, of which mutants can affect organ size and shape [40, 41]. Arahy. BC5R4P.1, was identified in the region II and is homologous to ARF2, which was reported to act as a target of BZR1 and negatively regulate seed size in *Arabidopsis* [42]. These four candidate genes together with other possible ones are worthy of further investigation to define their roles in peanut seed development.

## Conclusions

A new high density genetic map with 3866 SLAF and SSR loci was constructed based on the released cultivated peanut genome. Our findings demonstrated that this SLAF-based map was of high quality, and applicable for QTL mapping. A total of 27 QTLs regulating seed size and weight were identified in 4 environments, including two stable pleiotropic QTL regions, of which the QTL region on chromosome 2 was novel. These findings will facilitate the fine mapping and cloning of genes conditioning yield components and seed morphology traits. Genetic markers associated with these traits can be designed for molecular breeding of peanut with improved seed characteristics.

## Methods

### Plant materials and phenotyping

An F_2:6–8_ population of 181 RIL lines was derived from a cross between ‘Huayu36’ and ‘6–13’. The plant materials (including the parents and the RILs) used in this study were originally created by our laboratory and we have all the relevant rights to the materials. All materials were grown in the field in accordance with the local legislation. The ‘Huayu36’ cultivar is large-seeded with light red testa. The germplasm ‘6–13’ is small-seeded with light brown testa. The RIL population and its parental lines were planted in the experimental fields in Laixi (at N 36.86°, E 120.53°), Shandong Province (planted in May and harvested in September of 2017 and 2018); in Sanya (at N 18.65°, E 109.80°), Hainan Province (planted in November of 2017 and harvested in March of 2018); in Dongying (at N 37.46°, E 118.49°), Shandong Province (planted in May and harvested in September of 2018). The field experiments followed a randomized block design with three replications according to a previous study with a few modifications [57]. For each plot, 10 plants from each RIL line were grown 15-cm apart within a row, and an 85-cm gap was given between RILs. The parental lines were planted after every 20 rows as controls. Standard agricultural practices were applied for field management. Each plant was harvested individually at its maturity to prevent loss from over-ripening. Only eight plants in the middle of each row were used for trait measurement. Mature seeds determined by full size pods with dark inner carp color from each plant were measured for 100 seed weight, seed length, seed width and length to width ratio. The seed length and seed width were measured by using a parallel rule. The seed weight was taken on an electrical scale. The length to width ratio was calculated by dividing seed length by seed width. The mean values of each measured trait were used for phenotypic characterization. The phenotypic datasets of four seed traits in four environments are shown in an additional supporting file (Additional file 1:Table S1).

### Statistical analysis of phenotypic data

The mean value and standard deviation of each seed related trait for the parents and each RIL line were analyzed, and the Student’s t-test was conducted by SPSS statistics (IBM® SPSS® statistics 19). The normality of the population data was analyzed by the Kolmogorov-Smirnov test. According to the equation *h*^*2*^ = σ_g_^2^/(σ_g_^2^ + σ_ge_^2^/n + σ_e_^2^/nr), the broad-sense of heritability (*h*^*2*^) for seed related traits was calculated by ANOVA analysis with QTL IciMapping V4.1 (http://www.isbreeding.net/software/?type=detail&id=18). The σ_g_^2^, σ_e_^2^, and σ_ge_^2^ represented the variances of genotypes (G), environments (E) and interaction of genotypes and environments (G × E). The Pearson’s correlation coefficient between each two traits was obtained utilizing the SPSS statistics (IBM® SPSS® statistics 19).

### DNA extraction and SSR marker analysis

Young healthy leaves from the two parents and 181 RIL lines (F_2:6_) were collected at the seedling stage, frozen in liquid nitrogen, and stored at − 70 °C. Total genomic DNA was extracted by Plant Genomic DNA Kit (TIANGEN Biotech Beijing Co., Ltd). The concentration and quality of DNA were examined using electrophoresis on a 0.8% agarose gel and an ND–1000 spectrophotometer (NanoDrop, Wilmington, DE, USA). PCR reaction conditions for SSR analysis were: 3 min denaturation at 94 °C; 35 cycles of 1 min at 94 °C, 30 s at 55 °C, and 90 s at 72 °C; and then a final extension of 10 min at 72 °C, and storage at 4 °C. The PCR products were then separated on a 6% PAGE gel. 37 polymorphic SSR primers (Additional file 1: Table S2) were used to genotype the RIL population. SSR genotyping was performed as previously described [22].

### High-throughput sequencing and genotyping

After scanning the reference genome of peanut, the *Rsa*I and *Eco*RV-HF® (NEB, Ipswich, MA) enzymes were selected to digest the genomic DNA. The protocol of the SLAF-library construction has been previously described [19, 58]. The DNA fragments with indices and adaptors (SLAFs) of 314–414 bp were excised and diluted for pair-end sequencing using the Illumina HighSeq 2500 platform according to the Illumina sample preparation guide (Illumina, Inc., San Diego, CA) at the Biomarker Technologies Corporation (Beijing, China).

Low quality reads (quality score < 30, indicating a 0.1% chance of error) were discarded based on sequence similarity, and the SLAF paired-end reads were clustered by BLAT (−tileSize = 10 -stepSize = 5) [58]. Sequences with over 90% identity were grouped in one SLAF locus [59]. The SLAF in this study were classified into three types, non-polymorphic SLAF, polymorphic SLAF, and repetitive SLAF. Using the minor allele frequency (MAF) evaluation, alleles were defined in each SLAF. SNP calling was achieved according to GATK Best Practices (https://www.broadinstitute.org/gatk/guide/best-practices?bpm=DNAseq#variant-discovery-ovw). Polymorphic SNP markers were classified into eight segregation patterns (ab × cd, ef × eg, hk × hk, lm × ll, nn × np, aa × bb, ab × cc and cc × ab). The RIL population is obtained by a cross between two homozygous parents with genotype aa or bb. Thus, SNP markers fitting the aa × bb segregation pattern were used for genetic map construction. To ensure the quality of the genetic map, low quality SNPs were filtered out by the following rules [22]: SNPs with sequencing depth in parents ≤10-fold; complete degree ≤70%; SLAF with highly distorted segregation ratio from the expected 1:1 by Chi square (*χ*^2^) test; SLAF with more than 8 SNPs. The aa and bb represents the genotypes of ‘6–13’ and ‘Huayu36’, and related genotyping results of each RIL line by high quality markers are shown in an additional supporting file (Additional file 1: Table S3).

### High density genetic map construction

A linkage map was constructed [60] and the physical positions of markers were assigned in reference to the cv. Tifrunner genome sequence [34]. The high quality SLAF markers were grouped based on a pair-wise modified logarithm of odds (MLOD) scores. To ensure efficient construction of the high-density and high-quality map, High Map Strategy was used for ordering the SLAF and SSR markers and correcting genotyping errors within the chromosomes [61]. MSTMap was applied to obtain the marker orders of each group [62]. The SMOOTH algorithm was used for error correction [63], and a k-nearest neighbor algorithm was applied to the missing genotype imputation [64]. The Kosambi mapping function was applied to estimate the map distances [65]. The genetic positions of markers on each LG were displayed in an additional supporting file (Additional file 1: Table S4). In addition, a co-linearity map was generated to evaluate the map quality. Linkage group number corresponds to the chromosome number assigned by the Tifrunner reference genome.

### QTL mapping

The *R*/*qtl* package [66] was used to detect QTL and confirm the relationship between different markers around each QTL with the composite interval mapping method (CIM). The permutation test was repeated 1000 times with the LOD scores larger than 5% cutoff value. A logarithm of the odds (LOD) threshold value of 3.0 was applied to declare the presence of a QTL at 95% significance level. The positive and negative additive effect represented the favorable alleles were from ‘6–13’ and ‘Huayu36’, respectively.

### Functional annotation

Markers flanking the confidence intervals of the co-localized QTLs in 4 environments were selected to identify the candidate genes based on the genome sequences of the cultivated peanut (https://peanutbase.org/data/public/Arachis_hypogaea/Tifrunner.esm.TVDM/). For the functional annotation, gene content within the major QTL regions was compared with the Nr (nonredundent) protein sequences available at the UniProt database using the BLASTX algorithm. The associated hits were then searched for their respective Gene Ontology (GO) terms at www.geneontology.org [67]. Homologous protein sequences of putative candidate genes were obtained by the BLASTP algorithm against the Arabidopsis information resources (https://www.arabidopsis.org/index.jsp).

## Supplementary information

**Additional file 1: Table S1** Phenotypic datasets of four seed traits in four environments. **Table S2** Primer sequences of SSRs used in the present HDGM construction. **Table S3** Genotyping of the RIL lines by SLAF and SSR markers. **Table S4** Genetic positions of SLAF and SSR markers used for HDGM construction (XLS 10089 kb)

**Additional file 2: Table S5** SLAF-seq data of the parents and RIL population, **Table S6** Summary of SLAF and SNP markers in parents and RIL population, **Figure S1** Number of SNPs for different segregation pattern, **Figure S2** Colinearity analysis of each linkage group with the Tifrunner reference genome, **Figure S3** Comparison of protein sequences between the candidate genes and homologs in *Arabidopsis*. (XLS 402 kb)

**Additional file 3: Table S7** Gene content within the QTL region I. **Table S8** Gene content within the QTL region II. **Table S9** SNPs loacted in two stable and pleiotropic QTL regions (XLS 402 kb)

## Data Availability

The data sets supporting the results of this study are included in the manuscript and additional supporting files.

## Abbreviations

100SW: 100 seed weight
ADD: Additive effect
BR: Brassinosteroid
CI: Confidence interval
CIM: Composite interval mapping
GO: Gene ontology
HDGM: High density genetic map
L/W: Length to width ratio
LGs: Linkage groups
LOD: Logarithm of the odds
NGS: Next generation sequencing
PVE: Phenotypic variation explained
QTL: Quantitative trait locus
RIL: Recombinant inbred line
SL: Seed length
SLAF-Seq: Specific-locus amplified fragment sequencing
SNP: Single nucleotide polymorphism
SSR: Simple sequence repeat
SW: Seed width
