# Supplementary material for: QTL identification for seed weight and size based on a high-density SLAF-seq genetic map in peanut (Arachis hypogaea L.)
Source: BMC Plant Biol. 2019 Dec 3;19:537. doi: 10.1186/s12870-019-2164-5 (PMC6892246; doi:10.1186/s12870-019-2164-5)
Supplement: Supplementary file 2 — Additional file 2: Table S5 SLAF-seq data of the parents and RIL population, Table S6 Summary of SLAF and SNP markers in parents and RIL population, Figure S1 Number of SNPs for different segregation pattern, Figure S2 Colinearity analysis of each linkage group with the Tifrunner reference genome, Figure S3 Comparison of protein sequences between the candidate genes and homologs in Arabidopsis. (XLS 402 kb) [file 12870_2019_2164_MOESM2_ESM.doc]

###### Addition File 2

Table S5, S6; Fig. S1, S2, S3

**QTL identification for seed weight and size based on a high-density SLAF-seq genetic map in peanut (*Arachis hypogaea* L.)**

Shengzhong Zhang^1†^, Xiaohui Hu^1†^, Huarong Miao^1†^, Ye Chu^2^, Fenggao Cui^1^, Weiqiang Yang^1^, Chunming Wang^3^, Yi Shen^4^, Tingting Xu^1^, Libo Zhao^5^, Jiancheng Zhang^1^, Jing Chen^1^*

^1^ Shandong Peanut Research Institute, Qingdao, 266100, P. R. China

^2^ Department of Horticulture, University of Georgia Tifton Campus, Tifton, GA 31793 United States

^3^ State Key Laboratory for Crop Genetics and Germplasm Enhancement, Jiangsu Plant Gene Engineering Research Center, Nanjing Agricultural University, Nanjing 210095, P. R. China

^4^ Institute of Industrial Crops, Jiangsu Academy of Agricultural Sciences, Nanjing, 210014, P. R. China

^5^ Qingdao Agricultural Radio and Television School, Qingdao, 266071, P. R. China

^†^ Equal contributors.

* Corresponding author: Jing Chen

E-mail: mianbaohua2008@126.com

Tel: +86 532 87631512

**Table S5** SLAF-seq data of the parents and RIL population

| Sample ID | Total Reads | Total Bases | Q30 (%)^a^ | GC (%)^b^ |
| --- | --- | --- | --- | --- |
| Maternal | 48,509,032 | 9,700,417,448 | 94.77 | 36.94 |
| Paternal | 37,823,032 | 7,563,897,326 | 94.54 | 38.38 |
| RIL | 8,560,330 | 1,711,681,515 | 94.21 | 38.18 |

**^a^ Q30, percentage of sequenced bases with the quality score ≥30;**

**^b^ GC, percentage of guanine and cytosine in all four bases**

**Table S6** Summary of SLAF and SNP markers in parents and RIL population

| Sample ID | Number of SLAF | Number of Reads | Average Depth of SLAF | Number of SNP |
| --- | --- | --- | --- | --- |
| Maternal | 733,610 | 37,345,725 | 50.91 | 370,837 |
| Paternal | 693,570 | 36,295,674 | 52.33 | 356,281 |
| RIL | 506,417 | 8,207,746 | 16.13 | 293,244 |


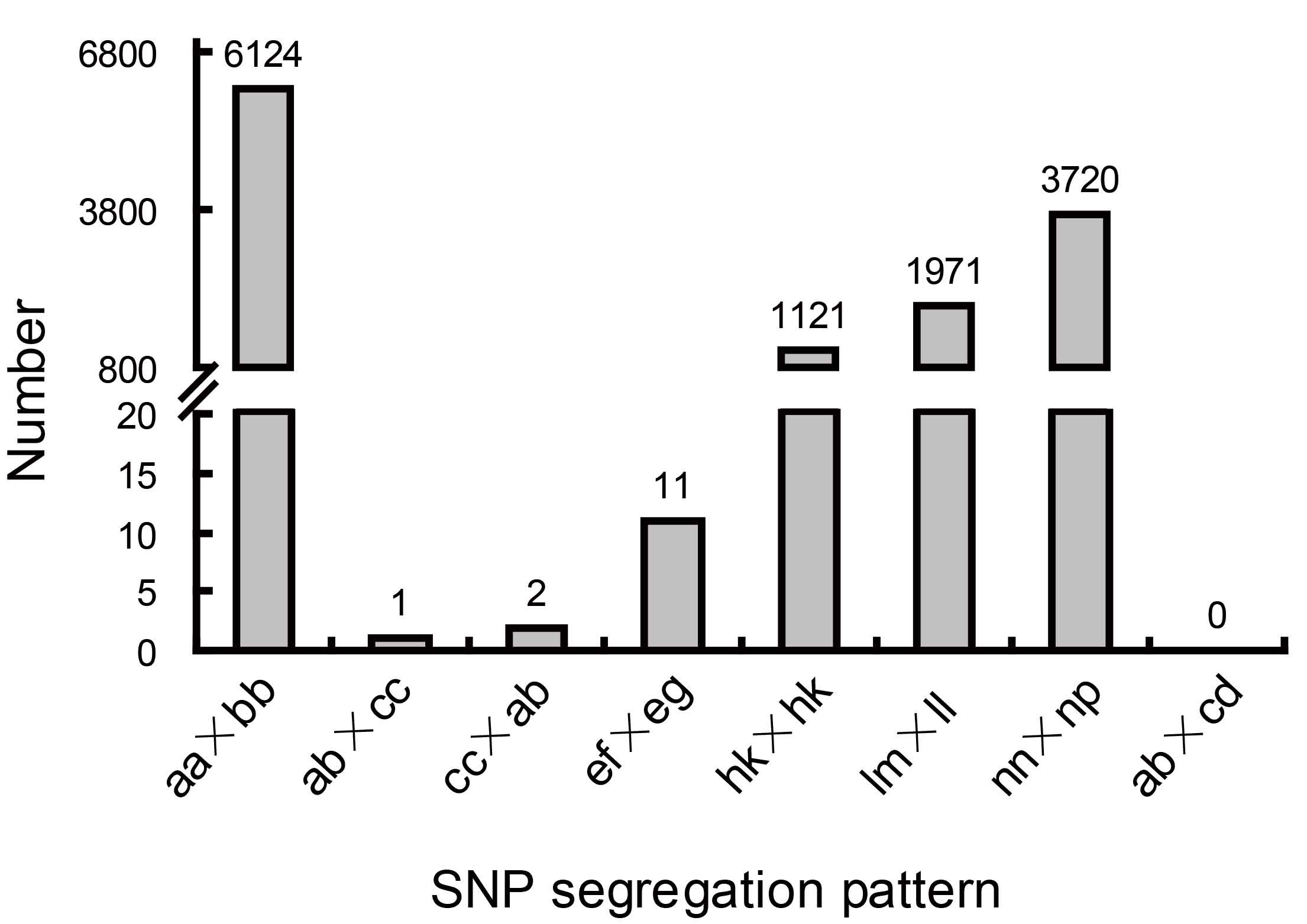


**Fig. S1** Number of SNPs for different segregation pattern.


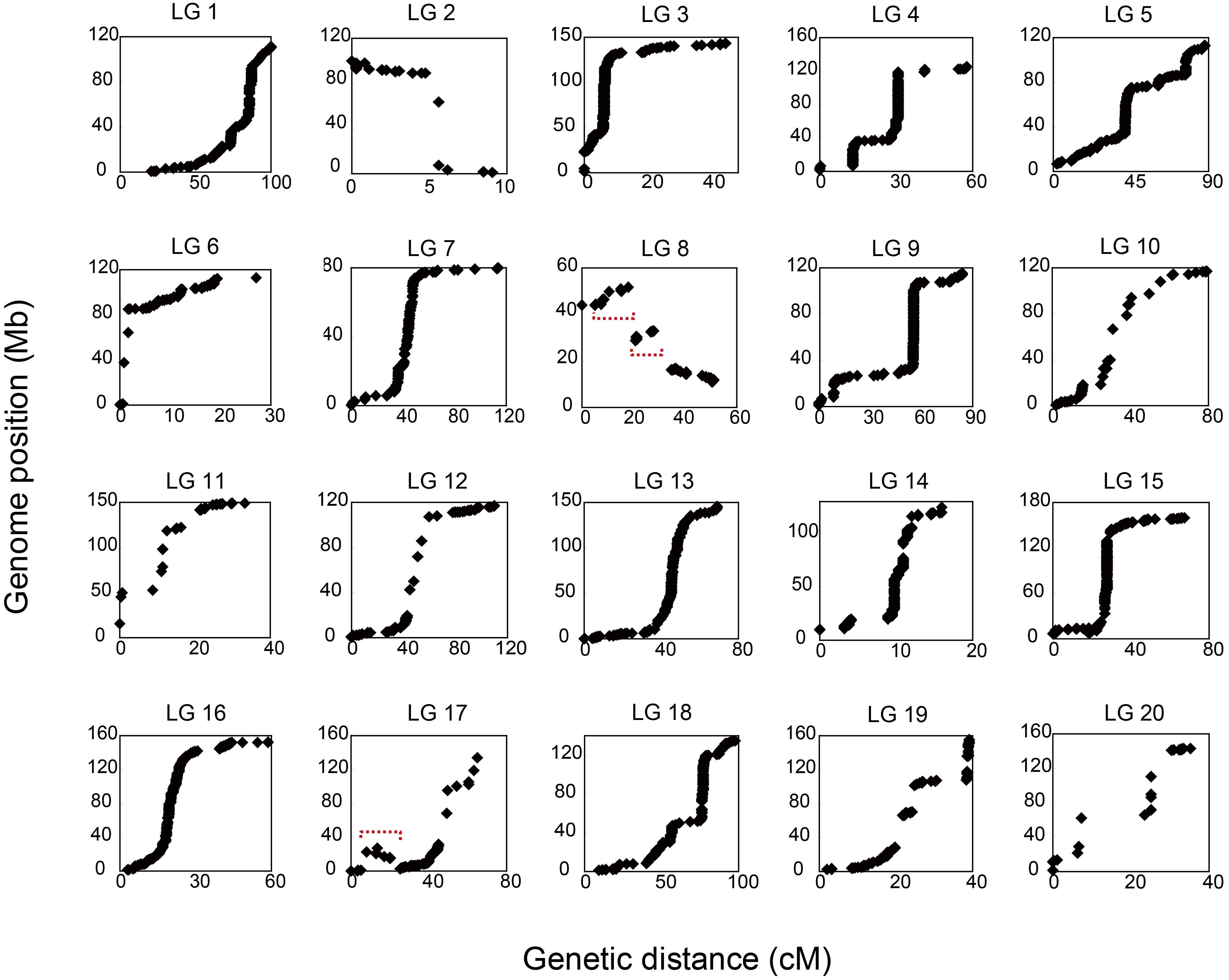


**Fig. S2** Colinearity analysis of each linkage group with the Tifrunner reference genome.

The x-axis scales the genetic distance of markers on each linkage group (LG). The y-axis represents the genome position of genetic markers based on Tifrunner reference genome. Three inversed segments were indicated by red dotted lines


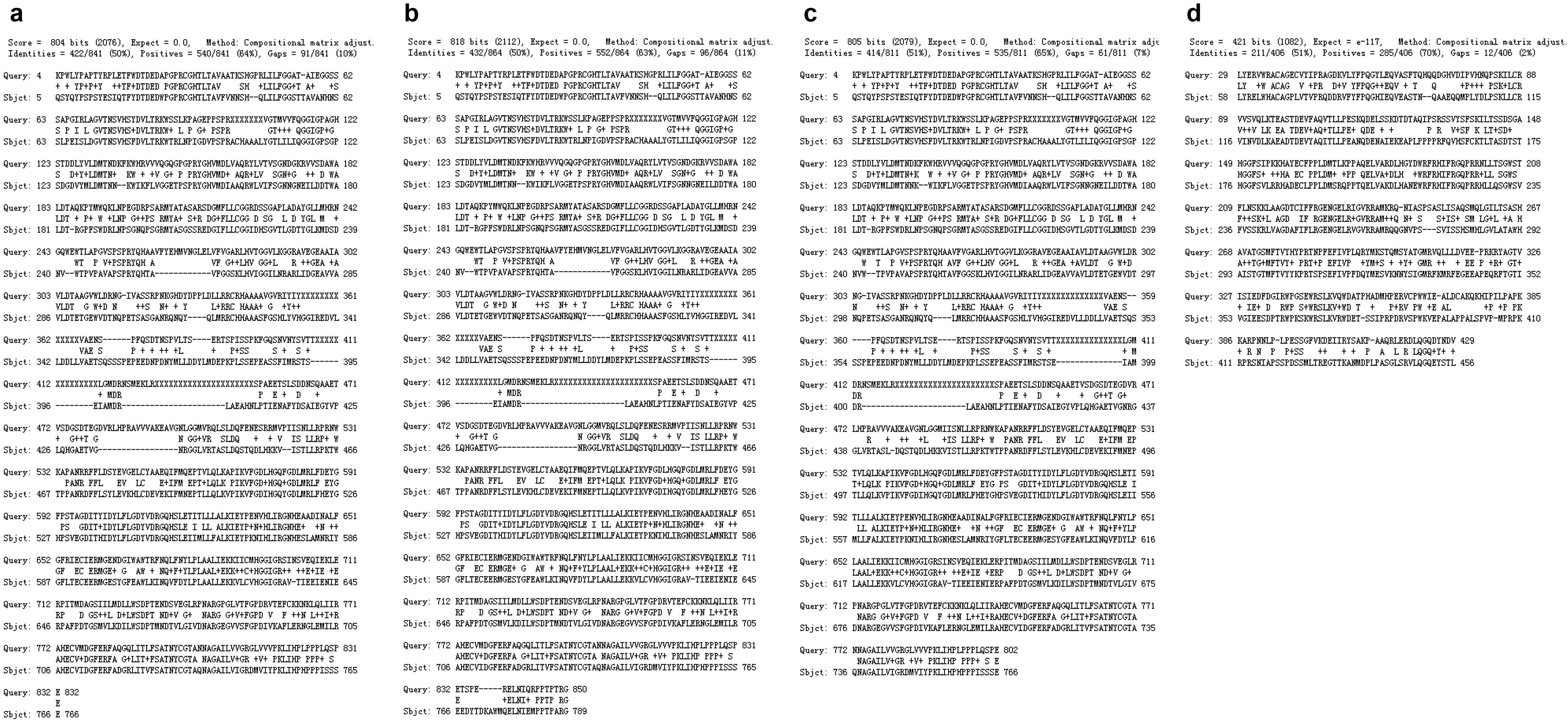


**Fig. S3** Comparison of protein sequences between the candidate genes and homologs in *Arabidopsis*.

(**a**), Sequence alignment of arahy.T43K8I.1 (Query) and BSU1 (Sbjct). (**b**), Sequence alignment of arahy.T43K8I.2 (Query) and BSU1 (Sbjct). (**c**), Sequence alignment of arahy.T43K8I.3 (Query) and BSU1 (Sbjct). (**d**) Sequence alignment of arahy. BC5R4P.1 (Query) and ARF2 (Sbjct)
